# Proteasome- and Calpain-Mediated Proteolysis, but Not Autophagy, Is Required for Leucine-Induced Protein Synthesis in C2C12 Myotubes

**DOI:** 10.3390/physiologia1010005

**Published:** 2021-11-08

**Authors:** Shelby C. Osburn, Christopher G. Vann, David D. Church, Arny A. Ferrando, Michael D. Roberts

**Affiliations:** 1 School of Kinesiology, Auburn University, Auburn, AL 36849, USA; 2 Duke Molecular Physiology Institute, Duke University School of Medicine, Duke University, Durham, NC 27708, USA; 3 Department of Geriatrics, Donald W. Reynolds Institute on Aging, University of Arkansas for Medical Sciences, Little Rock, AR 72205, USA

**Keywords:** leucine, muscle protein synthesis, muscle cells, proteasome

## Abstract

Muscle protein synthesis and proteolysis are tightly coupled processes. Given that muscle growth is promoted by increases in net protein balance, it stands to reason that bolstering protein synthesis through amino acids while reducing or inhibiting proteolysis could be a synergistic strategy in enhancing anabolism. However, there is contradictory evidence suggesting that the proper functioning of proteolytic systems in muscle is required for homeostasis. To add clarity to this issue, we sought to determine if inhibiting different proteolytic systems in C2C12 myotubes in conjunction with acute and chronic leucine treatments affected markers of anabolism. In Experiment 1, myotubes underwent 1-h, 6-h, and 24-h treatments with serum and leucine-free DMEM containing the following compounds (*n* = 6 wells per treatment): (i) DMSO vehicle (CTL), (ii) 2 mM leucine + vehicle (Leu-only), (iii) 2 mM leucine + 40 μM MG132 (20S proteasome inhibitor) (Leu + MG132), (iv) 2 mM leucine + 50 μM calpeptin (calpain inhibitor) (Leu + CALP), and (v) 2 mM leucine + 1 μM 3-methyladenine (autophagy inhibitor) (Leu + 3MA). Protein synthesis levels significantly increased (*p* < 0.05) in the Leu-only and Leu + 3MA 6-h treatments compared to CTL, and levels were significantly lower in Leu + MG132 and Leu + CALP versus Leu-only and CTL. With 24-h treatments, total protein yield was significantly lower in Leu + MG132 cells versus other treatments. Additionally, the intracellular essential amino acid (EAA) pool was significantly greater in 24-h Leu + MG132 treatments versus other treatments. In a follow-up experiment, myotubes were treated for 48 h with CTL, Leu-only, and Leu + MG132 for morphological assessments. Results indicated Leu + MG132 yielded significantly smaller myotubes compared to CTL and Leu-only. Our data are limited in scope due to the utilization of select proteolysis inhibitors. However, this is the first evidence to suggest proteasome and calpain inhibition with MG132 and CALP, respectively, abrogate leucine-induced protein synthesis in myotubes. Additionally, longer-term Leu + MG132 treatments translated to an atrophy phenotype. Whether or not proteasome inhibition in vivo reduces leucine- or EAA-induced anabolism remains to be determined.

## 1. Introduction

The maintenance of skeletal muscle mass relies upon the achievement of a net neutral protein balance, or the balance of protein synthesis and protein breakdown rates [1]. Protein synthesis is catalyzed in skeletal muscle by ribosomes that exist in the sarcoplasm and between myofibrils, and the mechanistic target of rapamycin complex 1 (mTORC1) is the signaling hub that leads to the formation of translation-competent ribosomes [2–4]. Three complex systems contribute to muscle proteolysis including the ubiquitin–proteasome pathway, autophagy/lysosomal proteolysis, and the calpain Ca^2+^-dependent cysteine proteases [5]. It is generally recognized that sustained proteolysis leads to muscle atrophy given that rodent and human studies show heightened proteolysis markers coincide with muscle loss [6–9]. However, there is evidence suggesting proteolysis is required for skeletal muscle homeostasis. For instance, ATG7 is involved in autophagosome formation, and reports from Masiero and colleagues show robust muscle atrophy occurs in *Atg7*-null mice [10,11]. Similar results have been reported in *Rpt3*-knockout mice [12]; notably, Rpt3 (or Psmc4) is a subunit of the 20S proteasome. Thus, while heightened and prolonged proteolysis lead to muscle atrophy, there is evidence to suggest a certain degree of proteolysis is required for muscle mass maintenance.

Interestingly, there is human evidence to suggest protein synthesis and breakdown rates are intricately linked processes that can be simultaneously stimulated by external stressors such as exercise [13,14]. Intricate cell culture work has also demonstrated that the active mTORC1 complex is localized at the lysosome [15], and others have shown that denervation-induced mTORC1 activation in rodent skeletal muscle is dependent on amino acids generated from proteasome-mediated degradation [16]. Moreover, it has been reported that the restriction of amino acids in vitro promotes protein degradation in order to prevent the depletion in intracellular amino acids [17]. The collective findings, as well as others [18], suggest a tight coupling of protein synthesis and breakdown.

L-leucine is an essential amino acid that has been shown to increase mTORC1 signaling [19] and translational efficiency [20]. Additionally, leucine administration reduces muscle proteolysis rates in vitro and in vivo [21]. These collective findings have led to the notion that L-leucine is anabolic as well as anti-catabolic. In spite of the evidence above suggesting a coupling between protein synthesis and breakdown, no study has determined whether proteolysis is required for leucine-induced muscle protein synthesis. Therefore, the purpose of this study was to determine if inhibiting different proteolytic systems in myotubes in conjunction with acute (1 and 6 h) and chronic (24 and 48 h) leucine treatments affected markers of anabolism. Notably, acute and chronic treatments were considered based on some of our prior in vitro work [22], as well as others’ in vitro work [19,23].

## 2. Methods

### 2.1. Cell Culture

Immortalized C2C12 murine muscle cells were purchased from the American Type Culture Collection (ATCC, Rockwille, MD, USA), and all incubations occurred at 37 °C in an atmosphere containing 5% CO2/95% air. Myoblasts (passage 2) were grown for two days in 6-well plates and cultured in growth media (GM) consisting of Dulbecco’s modified Eagle’s medium (DMEM; Corning, Corning, NY, USA) supplemented with 10% fetal bovine serum (FBS; Corning) and 1% penicillin/streptomycin (VWR International, Radnor, PA, USA). When cells reached 80–90% confluence, differentiation was induced by changing the GM to differentiation media (DM) which consisted of DMEM supplemented with 2% horse serum (VWR International). DM was replaced daily for four days. Thereafter (day 5 after the onset of differentiation), treatments occurred as described below.

### 2.2. Culture Treatments

**Figure 1.**
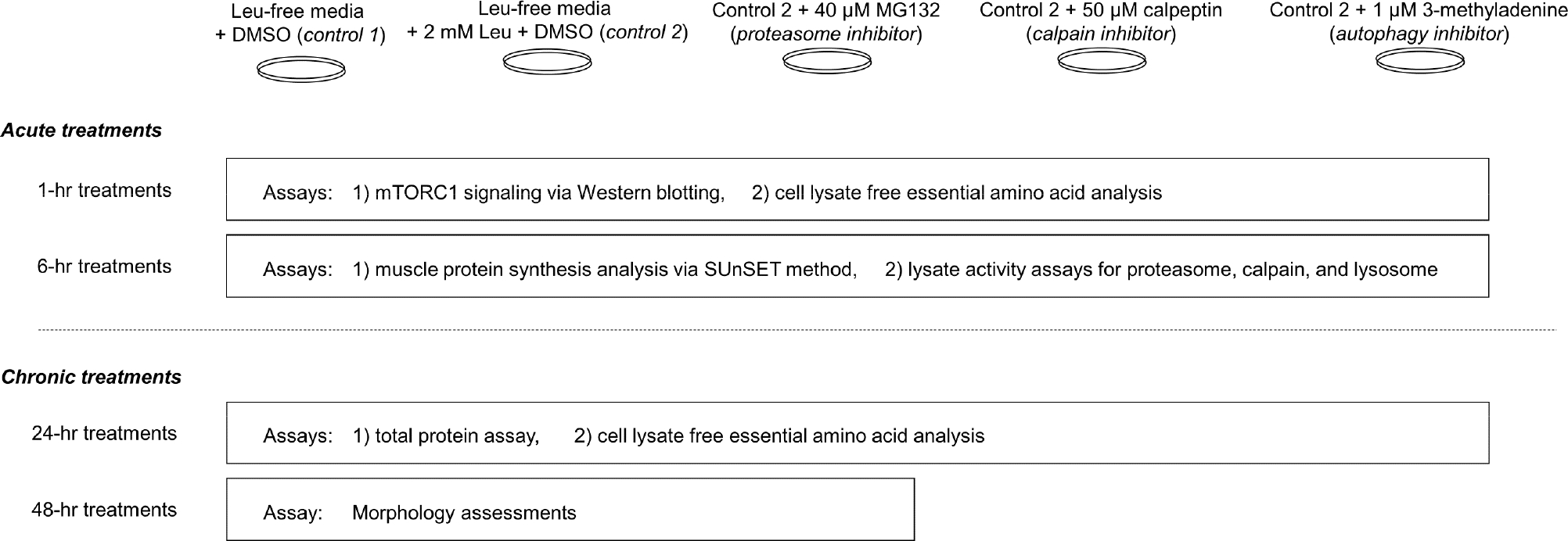
Study summary. This figure summarizes experiments performed in differentiated C2C12 myotubes. More in-depth descriptions of treatments and assays can be found in-text.

The study design is summarized in Figure 1 below.

The day of treatments, cells were rinsed with phosphate buffered saline (without calcium and magnesium; Corning), and were treated for 1, 6, or 24 h with serum and leucine-free DMEM (catalog #: 226-024; Crystalgen, Commack, NY, USA; Table 1) containing the following (*n* = 6 replicates per treatment): (a) DMSO vehicle (Leu-free CTL), (b) 2 mM leucine + vehicle, (c) 2 mM leucine + 40 μM MG132 (26S proteasome inhibitor; BML-PI102-0025; Enzo Life Sciences, Farmingdale, NY, USA), (d) 2 mM leucine + 2 mM 3-methyladenine (autophagy inhibitor; catalog #: AAJ64813-MB; Alfa Aesar, Ward Hill, MA, USA), and (e) 2 mM leucine + 50 μM calpeptin (calpain inhibitor; catalog #: 89161-562; Enzo Life Sciences). A dose of 2 mM leucine was chosen from Atherton et al. [19] who demonstrated that this dose elicited a significant increase in mTORC1 signaling, and 40 μM MG132 was chosen from Caron et al. [23] who demonstrated that this dose reduces proteolytic signaling in C2C12 myotubes. A dose of 50 μM calpeptin was chosen from Wei et al. [24] who demonstrated this dose reduces calpain activity in C2C12 myotubes. A millimolar 3MA concentration was chosen given that McMillan and Quadrilatero [25] demonstrated that 5 mM effectively inhibits LC3B production in myotubes without affecting PI3K signaling. Final DMSO concentrations per each treatment did not exceed 1% (*v/v*). For the second experiment, cells were rinsed with phosphate buffered saline before the 48-h treatment containing (a) DMSO only (Leu-free CTL), (b) 2 mM leucine + vehicle, and (c) 2 mM leucine + 10 μM MG132. For this experiment, we opted for a lower concentration of MG132, as long-term treatments with 40 μM were cytotoxic during the first attempt at this experiment. To demonstrate the effectiveness of 10 μM MG132 in reducing proteolytic signaling, we performed a 20S proteasome activity assay on the 48-h treatments. For reference, the amino acid content of Leu-free media is presented below.

### 2.3. Western Blotting for 1- and 6-h Treatments

Following treatments, cells were washed with PBS and lysed using plate scrapers and 250 μL of ice-cold cell lysis buffer (20 mM Tris-HCl (pH 7.5), 150 mM NaCl, 1 mM Na2EDTA, 1 mM EGTA, 1% Triton, 2.5 mM sodium pyrophosphate, 1 mM β-glycerophosphate, 1 mM Na3VO4, 1 μg/mL leupeptin; Cell Signaling, Danvers, MA, USA) pre-stocked with protease and Tyr/Ser/Thr phosphatase inhibitors. Slurries were placed in 1.7 mL microtubes and frozen at −80 °C until protein concentration determination.

Lysates were batch process-assayed for total protein content using a BCA Protein Assay Kit (Thermo Fisher; Waltham, MA, USA). Lysates were then prepared for Western blotting using 4x Laemmli buffer at 0.5 μg/μL. Thereafter, 15 μL of prepped samples were loaded onto 4–15% SDS-polyacrylamide gels (Bio-Rad, Hercules, CA, USA) and subjected to electrophoresis (180 V for 45–60 min) using pre-made 1x SDS-PAGE running buffer (VWR; Randor, PA, USA). Proteins were then transferred (200 mA for 2 h) to polyvinylidene difluoride membranes (Bio-Rad), Ponceau stained and imaged to ensure equal protein loading between lanes. Membranes were then blocked for 1 h at room temperature with 5% nonfat milk powder in Tris-buffered saline with 0.1% Tween-20 (TBST; VWR). Membranes containing 1-h treated samples were incubated with the following antibodies at a 1:1000 dilution in TBST for 48 h: rabbit anti-mouse phosphorylated mTOR (Ser2448) (catalog #: 5536; Cell Signaling), rabbit anti-mouse phosphorylated p70s6k (Thr389) (catalog #: 9205; Cell Signaling), and rabbit anti-mouse phosphorylated rps6 (Ser235/236) (catalog #: 2211; Cell Signaling). Membranes containing 6-h treated samples were incubated with the following antibodies at a 1:1000 dilution in TBST for 24 h: rabbit anti-mouse ubiquitin (catalog #: 3933; Cell Signaling), and mouse anti-puromycin (catalog #: MABE343; Millipore Sigma, Burlington, MA, USA). The following day, membranes were incubated with horseradish peroxidase-conjugated anti-rabbit or anti-mouse IgG (1:2000, Cell Signaling) in TBST with 5% BSA at room temperature for 1 h. Membrane development was performed using an enhanced chemiluminescent reagent (Luminata Forte HRP substrate; Millipore Sigma), and band densitometry was performed using a gel documentation system and associated densitometry software (ChemiDoc Touch, Bio-Rad, Shanghai, China). Densitometry values for all protein targets were normalized to Ponceau densities. These values were then normalized to Leu-free CTL values where the average for this treatment was set to 1.00, and data were expressed as relative expression units (REUs).

### 2.4. S Proteasome and Calpain Activity Assays for 6-h and 48-h Treatments

20S proteasome activity assays on 6-h and 48-h lysates were performed using commercially available fluorometric kits (catalog #: APT280; Millipore Sigma) as per the manufacturer’s instructions, which are similar to methods previously published by our laboratory [26]. Briefly, lysates (20 μL diluted 1:1 with diH_2_O) were loaded in duplicate onto black 96-well plates with the enzyme mix provided by the kit and incubated at 37 °C for 60 min. Fluorescence was then read using a microplate fluorometer (BioTek Synergy H1, Winooski, VT, USA) using 380 nm excitation and 460 nm emission settings. All fluorometric readings were divided by total protein loaded per well and expressed as relative fluorescent units (RFU) per μg protein. For 6-h treatments, the average coefficient of variation values for all duplicates was 23.5%. The high variability in the proteasome assay was in part due to the difference in readings in the MG132 treatment group. Without this group, the average coefficient of variation was 7.1% for this assay. For the 48-h treatments, the average coefficient of variation was 2.7% for this assay.

Calpain activity assays on 6-h lysates were performed using commercially available fluorometric kits (catalog #: ab65308; Abcam, Cambridge, UK). All fluorometric readings were divided by total protein loaded per well and expressed as relative fluorescent units (RFU) per μg protein. The average coefficient of variation values for all duplicates were 3.9%.

### 2.5. Protein Accretion Determination in 24-h Treated Cells

Cells were lysed as described above, and lysates were assayed for total protein levels per well were assayed in duplicate using a benchtop spectrophotometer (Nanodrop Lite; Thermo Fisher). The average coefficient of variation for duplicate readings was 0.92%.

### 2.6. Cytology for 48-h Treated Cells

Cells were stained for morphological assessment in the 48-h treatment experiment. Briefly, cells were washed with PBS before being fixed with 10% formalin for 10 min, rinsed in PBS again and permeabilized with 0.5% Triton X-100 for 5 min. Following permeabilization, cells were rinsed in PBS before blocking with SuperBlock for 30 min at room temperature. Following a PBS rinse, cells were incubated with a 1:50 dilution of a primary antibody against sarcomeric myosin (MF20 supernatant, Developmental Studies Hybridoma bank; 1:25 dilution) for 1 h at room temperature. Following primary incubation, cells were PBS rinsed and further incubated in a 1:100 dilution of a goat anti-mouse IgG conjugated to Alexa Fluor 488 secondary antibody (catalog #: A-11001; Invitrogen) for 1 h at room temperature. Cells were washed in PBS before being mounted with a diluted DAPI mounting media (catalog #: GTX30920; Genetex) with Vectashield (catalog #: H-1400-10; Vector Laboratories, Burlingame, CA, USA). Cells were imaged at 10× and analyzed for myotube morphology metrics using ImageJ (National Institutes of Health, Bethesda, MD, USA) similar to a previous study published by our laboratory [22]. This process first involved establishing a calibrator line to convert the arbitrary length units provided by the software to microns. Thereafter, the length and width of myotubes were obtained using this measurement function. Diameter measurements were obtained from 50 myotubes per treatment. Moreover, 3–5 width measurements (depending on the length of the myotube) were obtained and averaged together. A single length measurement was also obtained from these same 50 myotubes per treatment.

### 2.7. Free Essential Amino Acid Pool Analysis from Cell Lysates

Free essential amino acids were analyzed on 1-h and 24-h lysates using the internal standard technique. Briefly, 40 μL of lysate and internal standard were added to a micro centrifuge tube and vortexed. This mixture was then loaded onto Phree™ phospholipid removal columns (Phenomenex). Next, 300 μL of acetonitrile with 1% formic acid was added to the column. Samples were then centrifuged at room temperature at 500× *g* for 5 min, and subsequently placed in a speed-vacuum to dry. Samples were reconstituted in 50 μL of 1% sodium bicarbonate, and subsequently derivatized with 50 μL of fluorenylmethyloxycarbonyl at room temperature for 15 min. The reaction was stopped with 20 μL 0.05 N hydrogen chloride and analyzed by LC-MS/MS.

### 2.8. Statistics

All data were checked for normality using Shapiro–Wilks tests. Normally distributed data were compared using one-way ANOVAs with LSD post hoc tests. Non-normally distributed data were compared using Kruskal–Wallis tests with Mann–Whitney U post hoc tests. Select associations were also performed using Pearson’s correlations, and strong correlations were considered to be r values > 0.800. Data analysis was performed using SPSS (Version 26; IBM SPSS Statistics Software, Chicago, IL, USA). All data herein are presented as means ± standard deviation values.

## 3. Results

### 3.1. Effects of 1-h Treatment on mTORC1 Signaling

**Figure 2.**
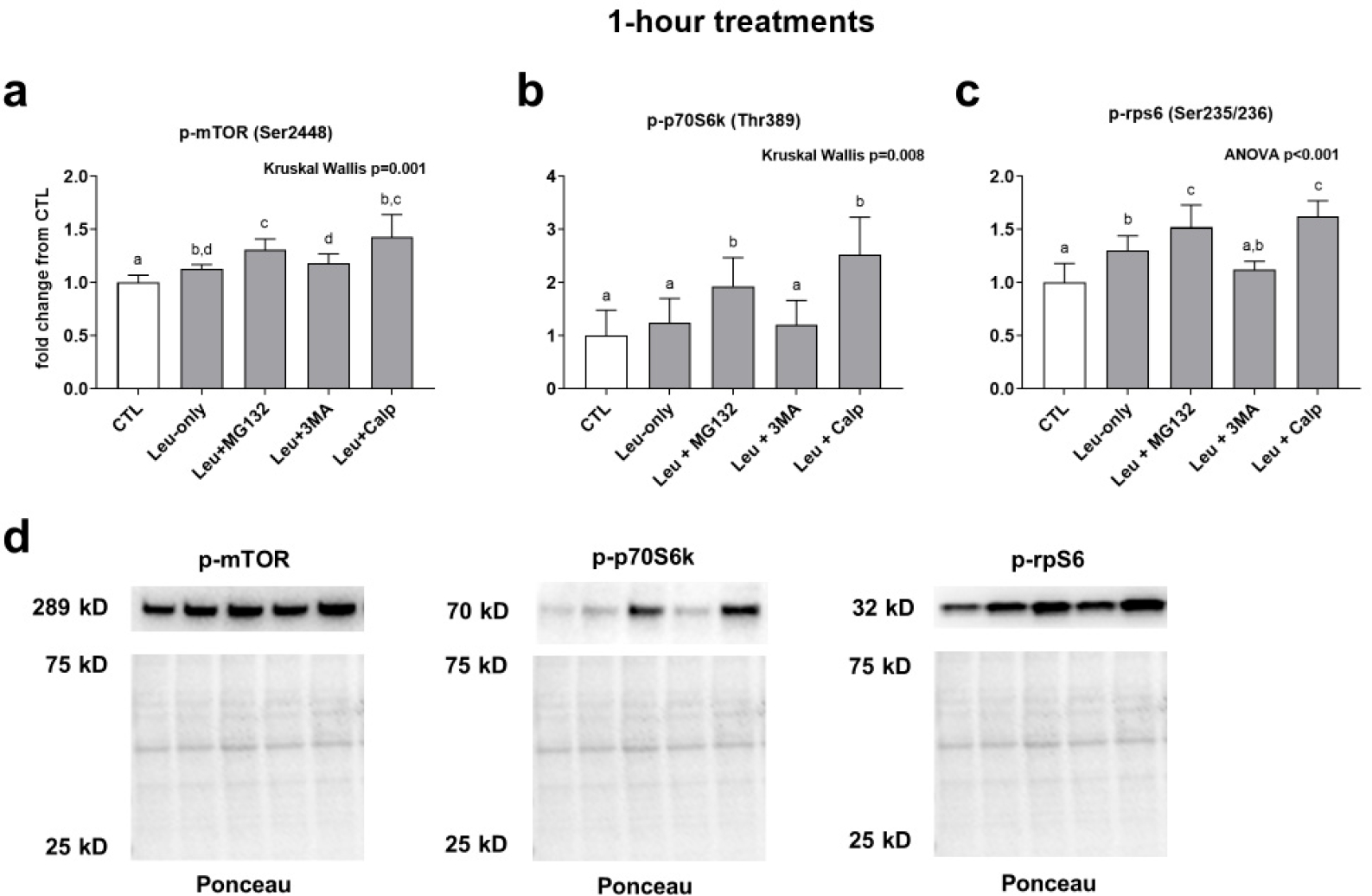
Effects of 1-h treatments on mTORC1 signaling. Phosphorylation of mTOR (**a**), p70S6k (**b**), and rps6 (**c**) in response to 1-h Leu-free CTL, Leu-only, Leu + MG132, Leu + 3MA, and Leu + CALP treatments. Panel (**d**) contains representative images of Western blots. Variables are presented in the treatment groups as a fold change from the Leu-free CTL. Bars that do not share the same letter indicate a significant difference between groups (*p* < 0.05). All data are presented as mean ± letter deviation values.

mTOR phosphorylation was significantly higher in all treatments compared to CTL. Furthermore, the Leu + MG132 treatment (but not the Leu + CALP or Leu + 3MA treatments) was significantly higher compared to the Leu-only treatment. (Figure 2a). p70s6k phosphorylation was significantly higher in the Leu + MG132 and Leu + CALP treatments compared to the CTL, Leu-only, and Leu + 3MA treatments (Figure 2b). Finally, rps6 phosphorylation followed a similar trend to mTOR phosphorylation. Specifically, Leu-only, Leu + MG132, and Leu + CALP treatments were significantly greater than the CTL treatment. Additionally, Leu + MG132 and Leu + CALP treatments were significantly higher than Leu-only treatments (Figure 2c). Collectively, these data suggest: (i) Leu-only treatments enhance various aspects of mTORC1 signaling, and (ii) Leu with calpain and proteasome inhibition further enhance mTORC1 signaling relative to Leu-only treatments.

### 3.2. Effects of 6-h Treatment on Proteasome Activity, Poly-Ubiquinated Protein Levels, and the Lc3-II/I Ratio

**Figure 3.**
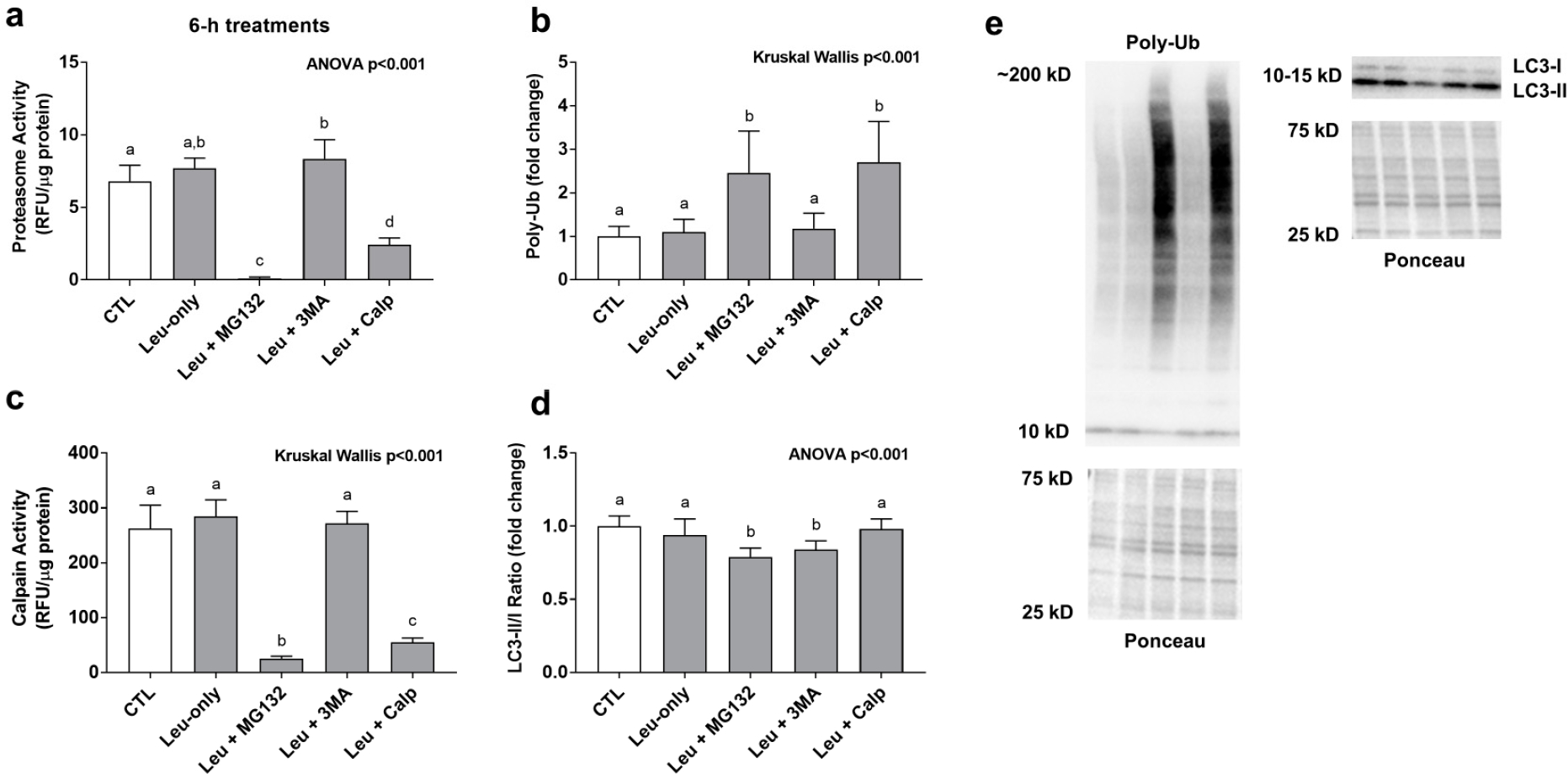
Effects of 6-h treatment on proteolytic system activity and poly-ubiquinated protein levels. These data are proteasome activities (**a**) poly-ubiquinated (Poly-Ub) protein levels (**b**), calpain activities (**c**), and the LC3II/I ratio (**d**) after 6-h treatments. Proteasome and calpain activities are presented as relative fluorescent units (RFU)/μg of protein. Poly-Ub (**b**) and LC3 II/I ratio (**d**) are presented in the treatment groups as a fold change from the Leu-free CTL. Panel (**e**) contains representative images from Western blotting. Bars that do not share the same letter indicate a significant difference between groups (*p* < 0.05). All data are presented as mean ± standard deviation values.

Leu + MG132 and Leu + CALP significantly decreased proteasome activity levels and calpain activity levels while significantly increasing poly-ubiquinated protein levels compared to all other treatments (Figure 3a–c). These findings suggest both inhibitors down-regulated cellular proteasome and calpain activity levels. The LC3-II/I ratio (an indicator of autophagic flux) was significantly lower with the Leu + MG132 and Leu + 3MA compared to all other treatments (Figure 3d).

### 3.3. Effects of 6-h Treatment on Muscle Protein Synthesis and 24-h Treatment on Protein Accretion

**Figure 4.**
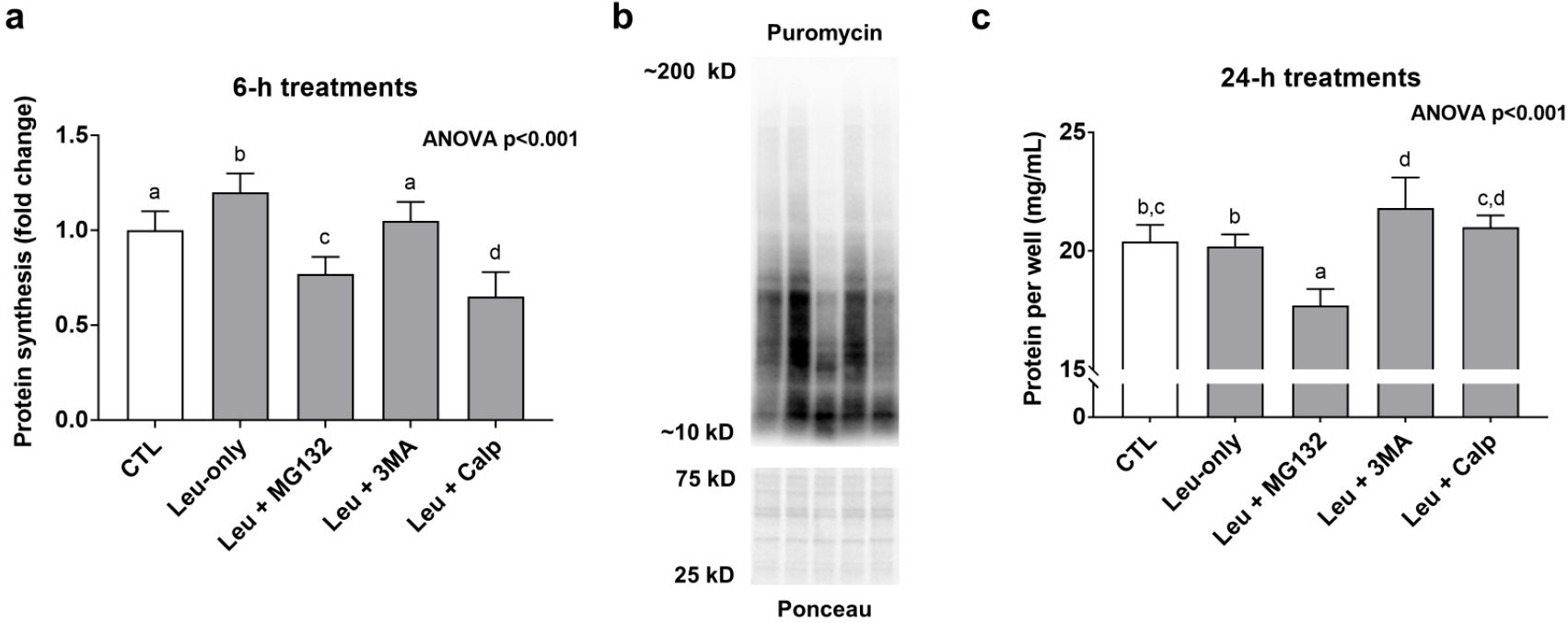
Effects of 6- and 24-h treatments on muscle protein synthesis and protein accretion. These data are protein synthesis levels after 6-h treatments (**a**), and total quantified protein per well after 24-h treatments (**c**). Panel (**b**) contains representative images from Western blotting. Bars that do not share the same letter indicate a significant difference between groups (*p* < 0.05). All data are presented as mean ± standard deviation values.

Leu-only treatments significantly increased MPS levels compared to CTL treatments suggesting a transient anabolic effect (Figure 4a). Interestingly, in spite of Leu + MG132 and Leu-CALP causing enhanced effects with mTORC1 signaling markers, both treatments presented MPS levels that were significantly lower than the other treatments (Figure 4a). Regarding longer-term treatment effects on muscle protein accretion, 24-h Leu + MG132 treatments reduced this metric (Figure 4c). Collectively, these data suggest Leu + MG132 and Leu + CALP treatments transiently reduced MPS levels, albeit a more prolonged atrophy effect (i.e., a loss of muscle protein following 24-h treatments) occurred only with Leu + MG132.

### 3.4. Associations between Protein Synthesis and Proteolysis Markers following 6-h Treatments

**Figure 5.**
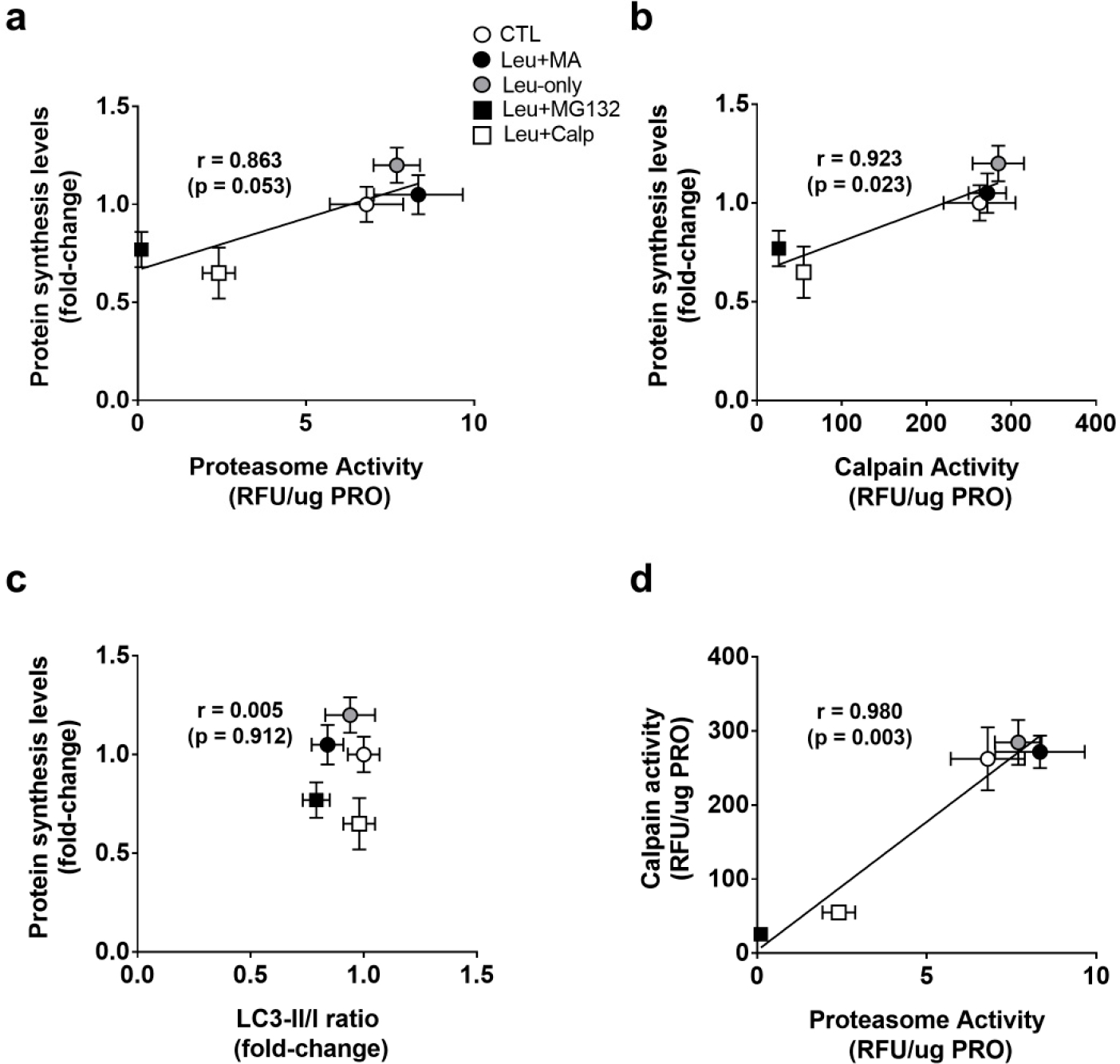
Select associations with 6-h treatments. Correlation analysis was performed on treatment means for protein synthesis levels and proteasome activity (**a**), protein synthesis levels and calpain activity (**b**), protein synthesis levels and LC3-II/I (**c**), and calpain and proteasome activity (**d**). All data are presented as mean ± standard deviation.

To explore whether protein synthesis levels and proteolysis markers exhibited a coupled relationship, we performed associations with 6-h treatments. When correlating 6-h treatment means for proteasome activities and protein synthesis levels, there was a strong positive correlation (r = 0.863, *p* = 0.053) (Figure 5a). When correlating 6-h treatment means for calpain activities and protein synthesis levels, there was also a strong positive correlation (r = 0.923, *p* = 0.023) (Figure 5b). When correlating 6-h treatment means for LC3-II/I ratios and protein synthesis levels, there was no significant association (r = 0.005, *p* = 0.912) (Figure 5c). Finally, when correlating 6-h treatment means for calpain and proteasome activities, there was a strong positive correlation (r = 0.980, *p* = 0.003) (Figure 5d). Collectively, these data suggest a tight coupling between MPS levels and calpain as well as proteasome levels. However, autophagic flux did not associate with any of these markers.

### 3.5. One-Hour and 24-h Treatment Effects on Cellular Free Pool Essential Amino Acid Concentrations

**Figure 6.**
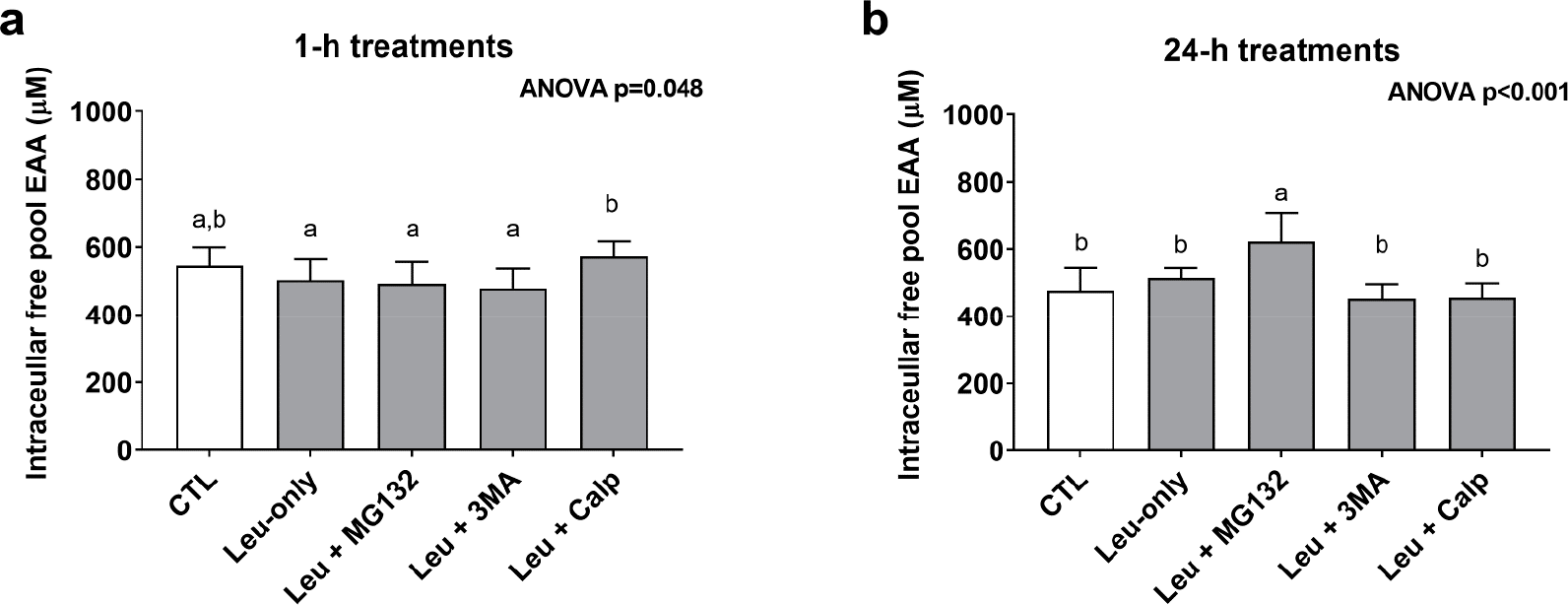
Intracellular free essential amino acid concentrations after 1- and 24-h treatments. These are concentration data for the intracellular free essential amino acid (EAA) pool after 1-h (**a**) and 24-h (**b**) treatments. Bars that do not share the same letter indicate a significant difference between groups (*p* < 0.05). All data are presented as mean ± standard deviation values.

In order to gain a better understanding as to how each treatment affected the free amino acid pool, cell lysates were analyzed for essential amino acid (EAA) concentrations from the 1-h and 24-h treatments. The one-way ANOVA for 1-h treatments was significant (*p* = 0.048); however, none of the treatments significantly differed from CTL or Leu-only (Figure 6a). The one-way ANOVA for 24-h treatments was also significant (*p* < 0.001). Interestingly, EAA concentrations were highest in Leu + MG132 24-h treatments compared to all other 24-h treatments (Figure 6b).

### 3.6. Effects of 48-h Treatment on Myotube Morphology

**Figure 7.**
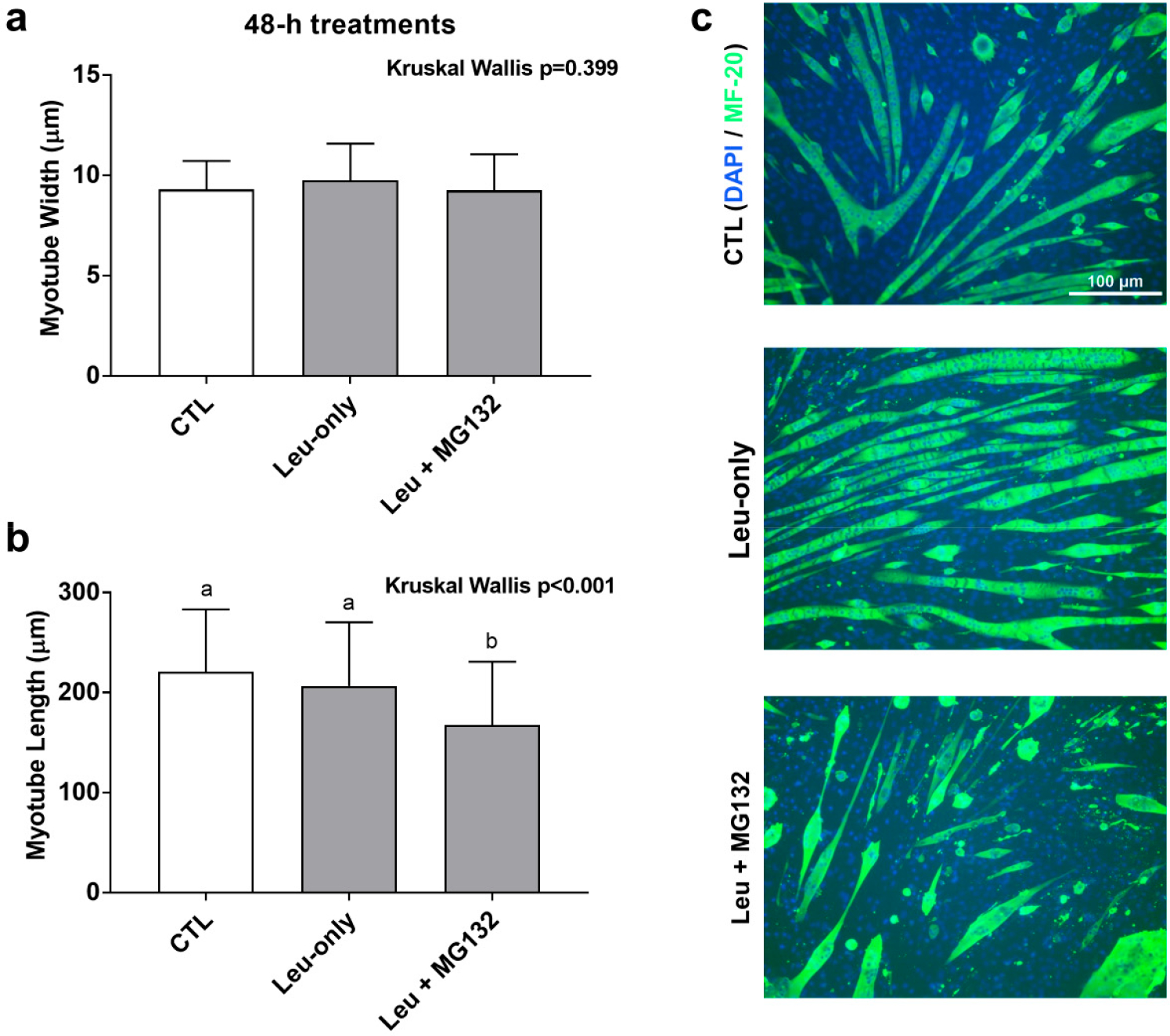
Effects of 48-h treatments on C2C12 myotube morphology. Changes in myotube width (**a**) and length (**b**) in response to a 48-h treatment on Leu-free CTL, Leu-only, and Leu + MG132. Representative images of the three treatments (**c**) were taken at 10× and analyzed using ImageJ. Bars that do not share the same letter indicate a significant difference between groups (*p* < 0.05). All data presented as mean ± standard deviation values.

Given that Leu + MG132 abrogated shorter- and longer-term anabolic responses relative to CTL and Leu-only treatments, we sought to further determine how proteasome inhibition affected cell morphology. A 48-h treatment was used to determine the effects of treatment on C2C12 morphology with regard to Leu-only and Leu + MG132 treatments. We had concerns that the dosage of MG132 used in the aforementioned experiments was cytotoxic with longer-term treatments, so we opted for a lower dosage for this experiment. To ensure the lower dosage adequately suppressed proteasome activity, a proteasome activity assay was performed on these treatments. Indeed, activity was lower in the Leu + MG132 treatment compared to CTL (*p* = 0.004) and Leu-only (*p* = 0.002) treatments, and there were no differences between CTL and Leu-only treatments (*p* = 0.261) (data not shown). There was no difference in myotube diameters between treatments (*p* = 0.399) (Figure 7a). However, myotubes were significantly smaller in the Leu + MG132 treatment compared to CTL (*p* < 0.001) and Leu-only (*p* = 0.001), but there was no difference between CTL and Leu-only (*p* = 0.288) (Figure 7b). Representative images of each treatment are shown in Figure 7c.

## 4. Discussion

Primary findings from this study include: (i) the allocated inhibitors effectively decreased the function of their respective proteolytic system, (ii) in agreement with prior literature, Leu-only stimulation increased markers of mTORC1 pathway activation and protein synthesis, (iii) although shorter-term Leu + MG132 and Leu + CALP treatments further enhanced markers of mTORC1 pathway activation compared to Leu-only, both treatments decreased protein synthesis compared to Leu-only and CTL treatments, (iv) the inhibition of the proteasome by MG132 was catabolic in spite of the co-treatment of cells with Leu, and (v) there appears to be a tight coupling between protein synthesis and the activities of the proteasome and calpain systems.

Perhaps the most interesting finding herein is that the co-treatment of myotubes with leucine and MG132 decreased markers of muscle anabolism (i.e., protein synthesis, protein accretion, and myotube size) in spite of acutely enhancing mTORC1 activity markers (1-h treatments) and elevating the intracellular EAA pool (24-h treatments). This suggests that the proper functioning of the proteasome is needed in order for leucine-induced protein synthesis to occur. Given that leucine is a potent stimulator of mTORC1 activity [14], and increased proteolysis stimulates atrophy [6], it seems logical that leucine stimulation along with proteasome inhibition would optimize net muscle protein balance and, therefore, enhance myotube hypertrophy. However, our data suggests otherwise. Within these experiments, proteasome-induced myotube atrophy was observed. Likewise, proteasome inhibition drastically abrogated leucine-induced increases in muscle protein synthesis. While it is difficult to reconcile these observations, we posit this effect may be due to multiple phenomena. First, proteasome inhibition may reduce the cellular free amino acid pool. Alternatively stated, the proteasome may function to enhance muscle protein synthesis by providing amino acids as substrates for the ribosome [18]. However, data in Figure 5 refute this hypothesis given that proteasome inhibition led to heightened levels of intracellular EAAs compared to other treatments. Thus, more likely scenarios include: (i) non-functioning proteasomes directly communicate and inhibit ribosomes through an unidentified mechanism, or (ii) MG132, in addition to inhibiting proteolysis, also directly inhibits protein synthesis. This former hypothesis is supported by various studies that collectively suggest the ubiquination process controls various aspects of ribosome function (reviewed in [27]). Conversely, the latter hypothesis agrees with other in vitro studies showing proteasome inhibition via MG132 disrupts the formation of polyribosomes and inhibits aspects of translation initiation [28,29].

There were other interesting findings herein. First, lysosome inhibition through 3MA did not disrupt, but rather enhanced, Leu-induced increases in both protein synthesis and 24-h protein accretion. Interestingly, the lysosome has been shown to be necessary for mTORC1 activity [12]. As stated prior, genetic mouse models have been used to demonstrate that chronic lysosome disruption elicits skeletal muscle atrophy [13]. It is difficult to determine why lysosome inhibition was anabolic herein. However, this effect may be transient and/or confined to in vitro effects, and warrants further investigation. While calpeptin treatments abrogated Leu-induced increases in protein synthesis, no effect was evident regarding protein accretion after 24-h treatments. Moreover, calpain inhibition did not lead to an accumulation of intracellular EAAs as seen with proteasome inhibition via MG132. Research has elucidated a dual role of calpains in protein metabolism through: (i) being involved with proteasome-dependent proteolysis, and (ii) inhibiting the Akt pathway and, thus, impairing muscle protein synthesis [30,31]. Our data certainly support the first of these prior findings given that the inhibition of the calpains through calpeptin reduced proteasome-dependent protein turnover while not affecting phosphorylation markers downstream of Akt. Although it is difficult to explain why calpeptin-induced decrements in protein synthesis did not ultimately affect protein accretion, it is notable that calpeptin did not reduce calpain or proteasome activities to the extent of MG132. Moreover, the effects that calpeptin had on reducing muscle protein synthesis may have been more transient compared to MG132, and this may have not affected protein accretion to a similar extent relative to proteasome inhibition.

### Experimental Considerations

Finally, it should be noted that all Western blot data herein were normalized to Ponceau stains, and not pan antibodies (in the case of mTORC1 markers) or a housekeeping marker (in the case of MPS data). While this is a limitation was mostly due to resource constraints, it should be noted that Ponceau S normalization for Western blotting is a widely practiced method, and can be more advantageous in situations where normalization to a housekeeping marker is not possible [32]. Additionally, signaling markers were interrogated only after 1-h treatments, and we have no reason to believe that this would alter protein levels of each interrogated mTORC1 target. Alternatively stated, treatments that facilitated increased level of phosphorylated mTORC1 markers likely did not do so by increasing overall levels of each respective protein after only 1-h treatments. Notwithstanding, our data should be viewed with this limitation in mind. Lastly, it should be noted that other compounds exist to inhibit the activities of the proteasome (e.g., proteasome inhibitor I and lactacystin) and calpains (e.g., calpain inhibitor IV and e-64-c). These compounds were not used herein. Given that MG-132 and calpeptin each inhibited proteasome and calpain activities, performing such experiments may have shed additional insight to our findings.

## 5. Conclusions

These findings highlight the necessary role that proteolytic systems, specifically Ub-mediated proteolysis, play in leucine-induced protein synthesis and anabolism. These data further support that an intricate relationship exists between protein synthesis and proteolytic systems in muscle cells. However, in vivo research is needed to further validate these findings.

## Supplementary Material

supplementary data

## Figures and Tables

**Table 1.** Amino acid content of Leu-free media.

Amino Acid	[μM] in Leu-Free Media
Arg	400
Cys	200
Gln	550
Gly	400
His	200
Iso	800
Leu	—
Lys	400
Met	200
Phe	400
Ser	400
Thr	80
Trp	80
Tyr	400
Val	800

Leu-free media data were constructed from product information provided by the vendor.

## Data Availability

The raw data will be provided with undue reservation by the corresponding author (mdr0024@auburn.edu) upon reasonable request.
