# Supplementary material for: Proteasome- and Calpain-Mediated Proteolysis, but Not Autophagy, Is Required for Leucine-Induced Protein Synthesis in C2C12 Myotubes
Source: Physiologia. Author manuscript; Available in PMC 2021 Dec 17. (PMC8681867; doi:10.3390/physiologia1010005)
Supplement: supplementary data [file NIHMS1759109-supplement-supplementary_data.docx]

**Supplemental data. Raw Western blots and raw data**

Note: the order from lanes 1-6 (left to right) are as follow:

Lane 1: Ladder

Lane 2: CTL

Lane 3: Leu-only

Lane 4: Leu+MG132

Lane 5: Leu+3MA

Lane 6: Leu+Calp

Lanes 7-11 and 12-16 then repeat the sequence of lanes 2-6

**1-hour treatments**

Gel 1 Ponceau


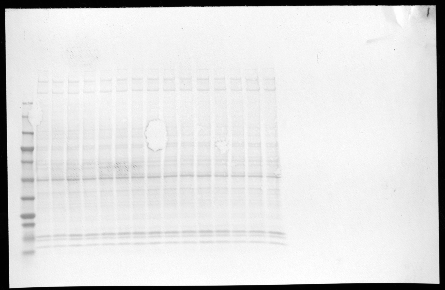


Gel 1 P-RPS6


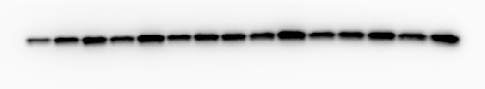


Gel 1 P-p70S6k


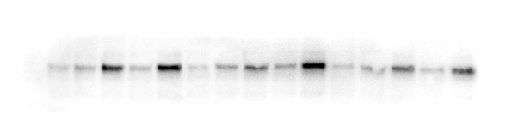


Gel 1 P-mTOR


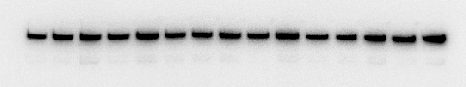


Gel 2 Ponceau


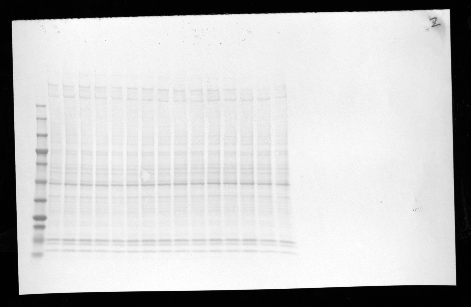


Gel 2 P-RPS6


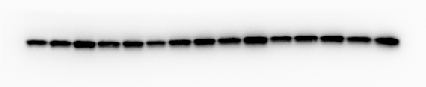


Gel 2 P-p70S6k


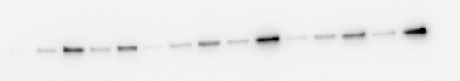


Gel 2 P-mTOR


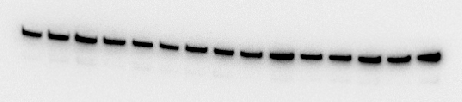


**6-hour treatment data**

Gel 1 Ponceau


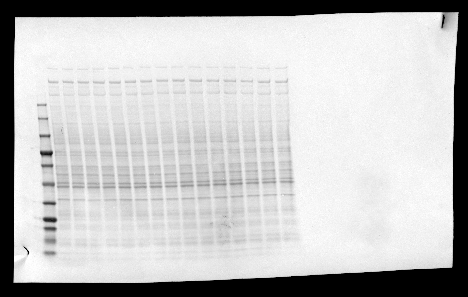


Gel 1 LC3 (bottom two rows)


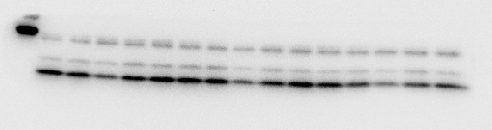


Gel 2 Ponceau


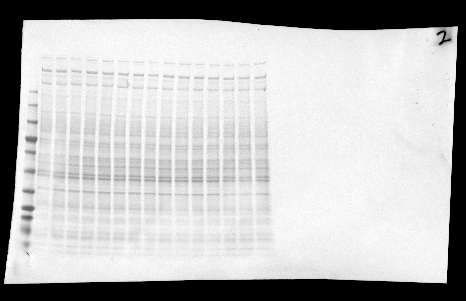


Gel 2 LC3 (bottom two rows)


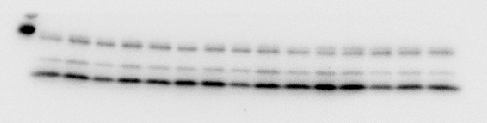


Gel 3 Ponceau


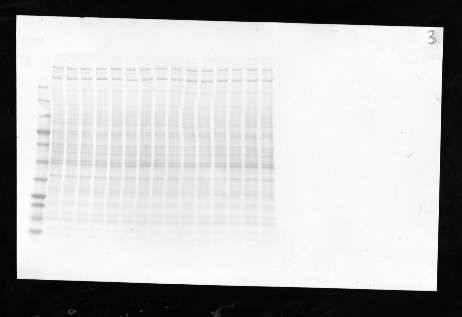


Gel 3 PolyUb (whole lane)


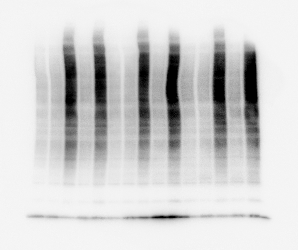


Gel 4 Ponceau (the wrong number is written on the gel as 3; disregard, as this is Gel 4)


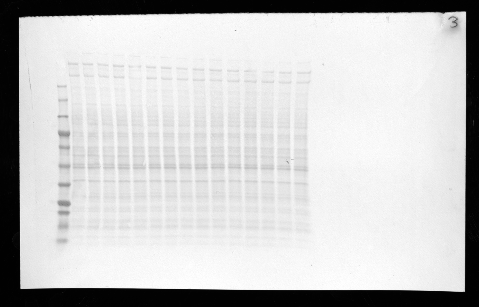


Gel 4 PolyUb (whole lane)


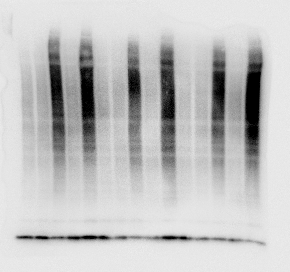


Gel 5 Ponceau


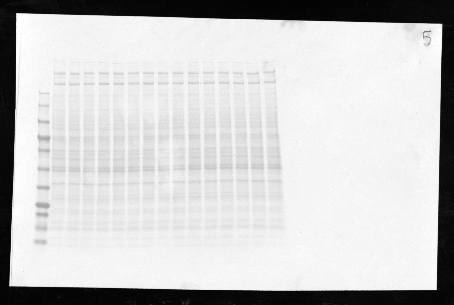


Gel 5 Puro (whole lane)


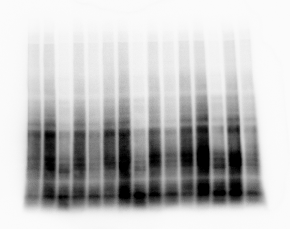


Gel 6 Ponceau


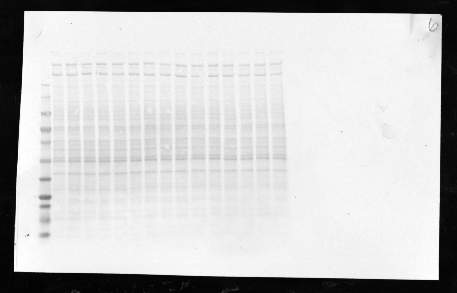


Gel 6 Puro (whole lane)


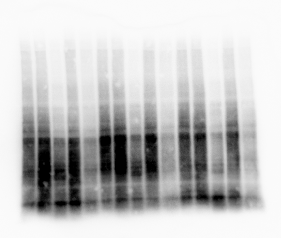


**Raw data (corrected for Ponceau and normalized to CTL)**

1-hr phosphorylated mTOR data

| Replicate # > | 1 | 2 | 3 | 4 | 5 | 6 |
| --- | --- | --- | --- | --- | --- | --- |
| CTL | 0.96 | 1.10 | 1.02 | 0.95 | 0.90 | 1.06 |
| Leu-only | 1.19 | 1.16 | 1.08 | 1.15 | 1.09 | 1.10 |
| Leu+MG132 | 1.37 | 1.25 | 1.40 | 1.35 | 1.13 | 1.37 |
| Leu+3-MA | 1.12 | 1.14 | 1.33 | 1.12 | 1.12 | 1.24 |
| Leu+Calp. | 1.42 | 1.47 | 1.58 | 1.02 | 1.51 | 1.58 |

1-hr phosphorylated p70s6k data

| Replicate # > | 1 | 2 | 3 | 4 | 5 | 6 |
| --- | --- | --- | --- | --- | --- | --- |
| CTL | 1.45 | 1.33 | 1.52 | 0.50 | 0.56 | 0.64 |
| Leu-only | 1.64 | 1.74 | 1.61 | 0.77 | 0.79 | 0.91 |
| Leu+MG132 | 2.65 | 2.25 | 2.28 | 1.68 | 1.24 | 1.49 |
| Leu+3-MA | 1.52 | 1.85 | 1.42 | 0.81 | 0.82 | 0.79 |
| Leu+Calp. | 2.94 | 3.44 | 2.43 | 1.31 | 2.40 | 2.61 |

1-hr phosphorylated rps6 data

| Replicate # > | 1 | 2 | 3 | 4 | 5 | 6 |
| --- | --- | --- | --- | --- | --- | --- |
| CTL | 0.83 | 1.15 | 1.16 | 1.11 | 0.73 | 1.02 |
| Leu-only | 1.18 | 1.45 | 1.30 | 1.45 | 1.25 | 1.13 |
| Leu+MG132 | 1.59 | 1.43 | 1.54 | 1.91 | 1.33 | 1.38 |
| Leu+3-MA | 1.06 | 1.19 | 1.08 | 1.04 | 1.26 | 1.12 |
| Leu+Calp. | 1.58 | 1.72 | 1.85 | 1.41 | 1.63 | 1.53 |

6-hr puromycin data (for MPS levels)

| Replicate # > | 1 | 2 | 3 | 4 | 5 | 6 |
| --- | --- | --- | --- | --- | --- | --- |
| CTL | 0.85 | 1.04 | 1.09 | 0.93 | 1.10 | 0.99 |
| Leu-only | 1.17 | 1.26 | 1.32 | 1.19 | 1.21 | 1.04 |
| Leu+MG132 | 0.77 | 0.75 | 0.83 | 0.86 | 0.80 | 0.61 |
| Leu+3-MA | 1.05 | 1.06 | 1.17 | 1.11 | 1.05 | 0.88 |
| Leu+Calp. | 0.76 | 0.78 | 0.70 | 0.66 | 0.53 | 0.44 |

6-hr poly-Ub protein data

| Replicate # > | 1 | 2 | 3 | 4 | 5 | 6 |
| --- | --- | --- | --- | --- | --- | --- |
| CTL | 1.03 | 1.22 | 1.32 | 0.80 | 0.87 | 0.76 |
| Leu-only | 1.25 | 1.44 | 1.37 | 0.90 | 0.82 | 0.79 |
| Leu+MG132 | 3.21 | 3.32 | 3.46 | 1.69 | 1.46 | 1.59 |
| Leu+3-MA | 1.38 | 1.60 | 1.47 | 1.01 | 0.74 | 0.90 |
| Leu+Calp. | 3.27 | 3.85 | 3.50 | 1.79 | 1.72 | 2.08 |

6-hr LC3-II/I ratio

| Replicate # > | 1 | 2 | 3 | 4 | 5 | 6 |
| --- | --- | --- | --- | --- | --- | --- |
| CTL | 1.07 | 0.90 | 1.03 | 0.93 | 1.03 | 1.05 |
| Leu-only | 0.99 | 0.91 | 0.81 | 0.84 | 1.04 | 1.07 |
| Leu+MG132 | 0.78 | 0.82 | 0.69 | 0.82 | 0.77 | 0.87 |
| Leu+3-MA | 0.83 | 0.89 | 0.75 | 0.86 | 0.79 | 0.94 |
| Leu+Calp. | 0.95 | 1.07 | 0.91 | 0.92 | 0.95 | 1.05 |
